# Polygenic risk for Alzheimer's disease shapes hippocampal scene-selectivity

**DOI:** 10.1038/s41386-019-0595-1

**Published:** 2020-01-02

**Authors:** Hannah L. Chandler, Carl J. Hodgetts, Xavier Caseras, Kevin Murphy, Thomas M. Lancaster

**Affiliations:** 1 Cardiff University Brain Research Imaging Centre (CUBRIC), School of Psychology, Cardiff University, Cardiff, CF24 4HQ UK; 2 MRC Centre for Neuropsychiatric Genetics & Genomics, School of Medicine, Cardiff University, Cardiff, CF24 4HQ UK; 3 CUBRIC, School of Physics and Astronomy, Cardiff University, Cardiff, CF24 3AA UK; 4 UK Dementia Research Institute, School of Medicine, Cardiff University, Cardiff, CF24 4HQ UK

**Keywords:** Genetic markers, Human behaviour, Predictive markers, Risk factors

## Abstract

Preclinical models of Alzheimer’s disease (AD) suggest *APOE* modulates brain function in structures vulnerable to AD pathophysiology. However, genome-wide association studies now demonstrate that AD risk is shaped by a broader polygenic architecture, estimated via polygenic risk scoring (AD-PRS). Despite this breakthrough, the effect of AD-PRS on brain function in young individuals remains unknown. In a large sample (*N* = 608) of young, asymptomatic individuals, we measure the impact of both (i) *APOE* and (ii) AD-PRS on a vulnerable cortico-limbic scene-processing network heavily implicated in AD pathophysiology. Integrity of this network, which includes the hippocampus (HC), is fundamental for maintaining cognitive function during ageing. We show that AD-PRS, not *APOE*, selectively influences activity within the HC in response to scenes, while other perceptual nodes remained intact. This work highlights the impact of polygenic contributions to brain function beyond *APOE*, which could aid potential therapeutic/interventional strategies in the detection and prevention of AD.

## Introduction

Alzheimer’s disease (AD) is the most common form of dementia and is a prominent cause of mortality in older populations. Genome wide association studies (GWAS) demonstrate that AD is highly polygenic, explained by the cumulative effect of thousands of single nucleotide polymorphisms (SNPs) [1, 2]. However, the impact of these SNPs on human brain function is still poorly understood, especially in large-scale cohorts of young adults where a more detailed characterisation of the aetiology of AD is needed to determine the effectiveness of possible therapeutic targets.

Population studies now suggest a genetic overlap between AD and common genetic variation that influences cognitive ability across the lifespan [3, 4], suggesting that AD risk alleles also influence the cognitive systems that support memory and intelligence. Several preliminary studies have explored the influence of individual single variants identified via AD GWAS such as loci within *APOE*, *CLU* or *PICALM* on brain function. This work broadly suggests that variation within these genes are associated with task-related brain activity, particularly in sub/cortical regions implicated in early AD associated Braakian atrophy including the hippocampus, entorhinal and cingulate cortices [5–11].

The hippocampus (HC) is one of the most studied anatomical regions in AD, associated with early and progressive atrophy in those at risk for developing the disease [12–14]. Evidence suggests that an increase in genetic risk for developing AD is associated with not only structural, but also functional brain alterations. Specifically, alterations in blood oxygen level dependent (BOLD) fMRI have been observed in AD patients and individuals who possess an *APOE*-ɛ4 allele [5, 6, 11]. Task-based fMRI paradigms have revealed alterations in BOLD response in individuals who possess a copy of the *APOE* ɛ4 allele. Yet very little attention has been paid to the wider cumulative impact of other common risk variants in AD. Specifically, little research has explored the relative contribution of the wider polygenic architecture that contributes to AD genetic risk on HC function.

Although the HC has largely been associated with episodic and spatial memory, emerging theoretical models of HC function highlight a critical role during complex scene processing across both memory and perception domains [15–17]. Though scene encoding is not considered solely a function of the HC [18–20], the HC is a key component in a wider circuit of brain regions affected in AD [21] where BOLD responses for scenes may be altered compared to other stimulus categories not typically associated with the HC (bodies, faces, tools). While these functions are classically associated with specific brain structures, including the HC, it is still unclear if BOLD in response to these types of tasks is altered in those with a genetic predisposition to developing AD, and if this contribution is based on the presence of *APOE* ɛ4 or the broader cumulative impact of common AD-related genes. Demonstrating a relationship between genetic risk and HC BOLD in response to scenes will provide grounding evidence that the wider, cumulative effect of multiple common risk genes should be considered in relation to brain function. This will help to inform preclinical models that predict the development of AD. Although associations between spatial/scene processing and genetic risk for AD (such as *APOE* ɛ4 dosage [22]) have been documented, working toward a larger polygenic model will provide more power for assessing the broader relationship between genes that confer risk and brain function.

In the present study, we combine imaging and genetic data from healthy individuals recruited by the Young Human Connectome Project (YA-HCP) to investigate the combined impact of AD common risk alleles on HC activity as assessed with BOLD during scene encoding in young healthy individuals (aged 22–35). Based on prior work that evidences hyperactivity in pre-symptomatic individuals at risk of developing AD [23], we predict a positive association between scene related HC BOLD and genetic risk assayed by AD-PRS (excluding the *APOE* region) in this young healthy cohort. In this study, AD common allele risk is measured using (i) an AD-polygenic risk score (AD-PRS) (excluding the *APOE* region with chromosome 19) and (ii) the presence/absence of APOE ɛ4. To assay scene encoding we use the perceptual categories embedded within the YA-HCP n-back paradigm, averaging across memory load. We suggest that scene processing provides a locus capable of probing HC function in those at genetic risk for developing AD. We perform post-hoc follow up studies to (i) search for AD-PRS and *APOE* effects within hippocampal voxels during scene encoding and (ii) repeat this analysis within an expanded search space (whole-brain level). We anticipate that these observations will elucidate the impact of the common genetic risk architecture for AD on hippocampal and/or scene encoding BOLD in asymptomatic individuals. Within this study we aim to provide grounding evidence for the wider cumulative effect of multiple common AD risk genes to inform preclinical models that predict the development of AD.

## Methods

### Sample

Participants were drawn from the March 2017 public data release from the YA-HCP; *N* = 1200. Participants were aged from 22 to 35, for all inclusion/exclusion criteria see Van Essen et al. [24]. Briefly, the study excluded individuals with a history of psychiatric disorder, substance abuse, neurological or cardiovascular disease and associated hospitalisation or long-term (>12 months) pharmacological/behavioural treatment. Each participants provided written informed consent. All subject recruitment procedures and informed consent forms (including consent to share de-identified data), were approved by the Washington University in St. Louis Institutional Review Board (IRB). Participants were excluded from the current analyses if they lacked good-quality structural magnetic resonance imaging data (for registration purposes), or had missing relevant interview/questionnaire data (Table 1; for demographic details). The complete imaging sample size, including related individuals was *N* = 608, which has 80% power to detect relatively small effects (*R*^2^ > 0.012). For further information on the HCP pedigree/kinship structure see http://www.humanconnectome.org/storage/app/media/documentation/s1200/HCP_S1200_Release_Reference_Manual.pdf.

**Table 1:** Descriptive/demographic statistics for individuals included in final mixed linear regression models.

	Combined sample		*APOE* ɛ4 (−) *N* = 462		*APOE* ɛ4 (+) *N* = 146		*P*
Gender	*M* = 285/*F* = 323		*M* = 215/*F* = 247		*M* = 70/*F* = 76		0.84
	Mean	SD	Mean	SD	Mean	SD	
Age	28.93	3.63	28.76	3.61	29.47	3.69	0.05
SSAGA_Education	15.04	1.70	15.02	1.73	15.10	1.61	0.60
Frame-wise displacement	0.09	0.03	0.09	0.03	0.09	0.04	0.57
HC Volume (mm^3^)	4497.00	440.73	4485.72	437.79	4533.67	449.53	0.26

X^2^ = chi squared test for gender. All other demographics were tested via two-sample *t*-test

*M* mean, *SD* standard deviation

### Genotyping and AD-PRS creation

All YA-HCP data are publicly available, including genome-wide genotype data to be distributed through dbGAP. Quality controls was implemented in PLINK v1.9 [25]. Briefly, single nucleotide polymorphisms (SNPs) were excluded where the minor allele frequency was less than 1%, if the call rate was less than 98%, or if the *χ*^2^ test for Hardy-Weinberg equilibrium had a *P* value less than 1 × 10^−4^. Individuals were excluded for ambiguous sex (genotypic sex and phenotypic sex not aligning or genotyping completeness less than 97%. A total of 1,137,480 variants and 1119 individuals were considered for AD-PRS creation. To account for the extended twin design, we created a kinship matrix and derived the top 20 principle components (PCs) from the linkage disequilibrium pruned data set and included PCs in all AD-PRS analysis. Participants that did not match the race of the discovery sample GWAS were excluded from the analysis. AD-PRS were created using the ‘score’ command in PLINK v1.9 [26] via the PRSice v1.25 software package [27]. AD genetic risk was estimated using publicly available results data from an international GWAS [2]. SNPs (single nucleotide polymorphisms) were removed from the AD GWAS data if they had a low MAF (minor allele frequency < 0.01) and were subsequently pruned for LD using a stringent clumping strategy (–r2 0.1, –kb 500). As SNPs may be correlated, pruning the SNPs ensured all SNPs included in each AD-PRS model were independent. The entire *APOE* region (from-kb 44,400 to -kb 46,500) was removed during the consideration of alleles for AD-PRS. We chose initially choose a liberal AD-PRS at *P*_T_ < 5 × 10^−1^, previously been shown to be most predictive of AD in case-control studies [28, 29]. In a post-hoc analysis, we create a series of P-threshold across a range of thresholds that have also been shown to associate with AD neuroimaging phenotypes [30, 31]. Individual *APOE* status was determined by the absence/presence of an ɛ4 allele calculated via rs7412 and rs429358.

### Description of fMRI paradigm

The scene—encoding BOLD signal was measured via fMRI during a scene-localiser paradigm, embedded in an *n*-back working memory task, as previously described [32]. Participants completed an *n*-back (0 and 2 back) task with multiple visual conditions (scenes, body parts, tools, faces). Within each run, 1/2 of the blocks use a 2-back working memory task (respond ‘target’ whenever the current stimulus is the same as the one presented two stimuli previously) and 1/2 use a 0-back working memory task (a target cue is presented at the start of each block, and the person must respond ‘target’ to any presentation of that stimulus during the block). A 2.5 s cue indicates the task type (and target for 0-back) at the start of the block. Each of the two runs contains 8 task blocks (10 trials of 2.5 s each, for 25 s) and 4 fixation blocks (15 s each). On each trial, the stimulus is presented for 2 s, followed by a 500 ms ITI. Each block contains 10 trials, of which 2 are targets, and 2–3 are non-target lures (e.g., repeated items in the wrong *n*-back position, either 1-back or 3-back). Our principal contrast was BOLD averaged across *n*-back load for scenes > average (faces, bodies and tools).

### BOLD parameter acquisition

BOLD parameter estimate acquisition YA-HCP sample Individual, pre-processed task-fMRI (tfMRI) directories for the n-back task were downloaded from the WUMinn HCP Data-1200 Subjects + 7 T data release at https://db.humanconnectome.org/, package type = MSMSulc- + MSM-All. All preprocessing steps and preliminary analysis were performed in FSL [33] and have previously been reported [32]. Briefly, the HCP ‘fMRIVolume' pipeline performs gradient unwarping, motion correction, fieldmap unwarping and grand mean intensity normalisation on the four-dimensional (4D) time series. These volumes are segmented (Brain Boundary Registration), registered to the T1 anatomical volume using nonlinear transformation (FNIRT) and warped to standard (MNI152) space. Parameter estimates were estimated for a pre-processed time series using a general linear model (GLM) using FMRIB's improved linear model (FILM) with autocorrelation correction. Predictors were convolved with a double gamma canonical hemodynamic response function to generate regressors. Temporal derivatives of each regressor were added to the GLM as covariates of no interest. Parameter estimates (BOLD) for the principal contrast (scenes > other) were available for 902 individuals from the whole sample (*N* = 1206). We chose this contrast to establish potential relationships specifically with scenes, rather than any other visual stimuli within the paradigm to estimate and probe HC BOLD. Using a custom series of wb_commands from the connectome workbench (https://www.humanconnectome.org/software/connectome-workbench.html), we then extracted BOLD parameter estimates from individual subject-pre-processed data (scenes > other) for the bilateral hippocampus as defined by the Harvard-Oxford Subcortical Structural Atlas.

### Statistical inferences

On the basis of prior recommendations [34], we first employed linear mixed effects models, estimated in R 3.3.2 (https://www.r-project.org/) using the lme package [35]. Both AD-PRS and *APOE* ɛ4 were entered into the model as fixed effects along with covariates including sex, age, education level (SSAGA_Educ), and head motion (average frame-wise displacement across the task). To account for population stratification, we also included 20 principle components (from a version of the genotype dataset which had been pruned for linkage disequilibrium) as covariates. To account for the familial structure in the sample, a sparse kinship matrix was included in each of the seven LME models using the ‘lme4qt’ package [21]. Minor allele frequencies were comparable between our training and test data and our AD-PRS model was normally distributed suggesting minimal between sample heterogeneity [36]. Sample outliers were removed from BOLD parameter estimates using the interquartile range (IQR) outlier labelling rule (1.5 × IQR (*Q*3–*Q*1)) as previously described [37]. After the removal of statistical outliers, HC BOLD was normally distributed (Shapiro test, *P* > 0.05). This method of outlier detection was also used for all post-hoc analysis. The AD-PRS predictors did not show evidence of collinearity with other covariates (variance inflation factor > 1.15 in all cases).

### Voxel-wise analysis

In order to ascertain regional hippocampal effects, we then performed voxel-wide search of the hippocampus using permutation analysis of linear models (PALM, [38]). Contrasts of parameter estimates for [scenes > other] were merged for all participants (*N* = 606) into a 4D cifti file. A separate 4D image of the bilateral hippocampus parameter estimates (for scenes > other) was then created using the HCP command line toolbox “wb_command”. The corresponding design matrix included the same regressors as the average hippocampal BOLD analysis including: AD-PRS, *APOE* status, age, sex, education, frame-wise displacement and the 20 genetic principle components to account for population stratification. To control for the HCP kinship/pedigree structure during tail approximation permutation testing (*N* = 5000) [39], we created exchangeability blocks [40] for the participants using code available at https://raw.githubusercontent.com/andersonwinkler/HCP/master/share/hcp2blocks.m and corrected for the family wise error using threshold free cluster enhancement (TFCE: [41]).

### Negative control analysis

We further aimed to quantify the specificity of AD genetic risk—HC BOLD relationships. We therefore extracted the average parameter estimates from (i) two other bilateral cortical regions in the scene > other network including a contiguous cluster of the (a) parahippocampal gyrus—transverse occipital sulcus (PHG-TOS) and (b) the retrosplenial cortex (RSC) and (ii) bilateral key cortical region for (c) body > other network (extra-striate body area: EBA); (d) face > other network (fusiform face area; FFA) and (e) object > other (lateral occipital cortex: LOC) and repeated the linear mixed effect model analysis [42–47]. All clusters were derived from a 1 sample *t*-test of the combined sample with a stringent threshold to delineate individual clusters (*t*-statistic > 15.5, in all cases). Together, these control analyses permitted us to quantify the specificity of the (i) region and (ii) the contrast.

## Results

### Whole group effects of BOLD for scenes > other

**Fig. 1:**
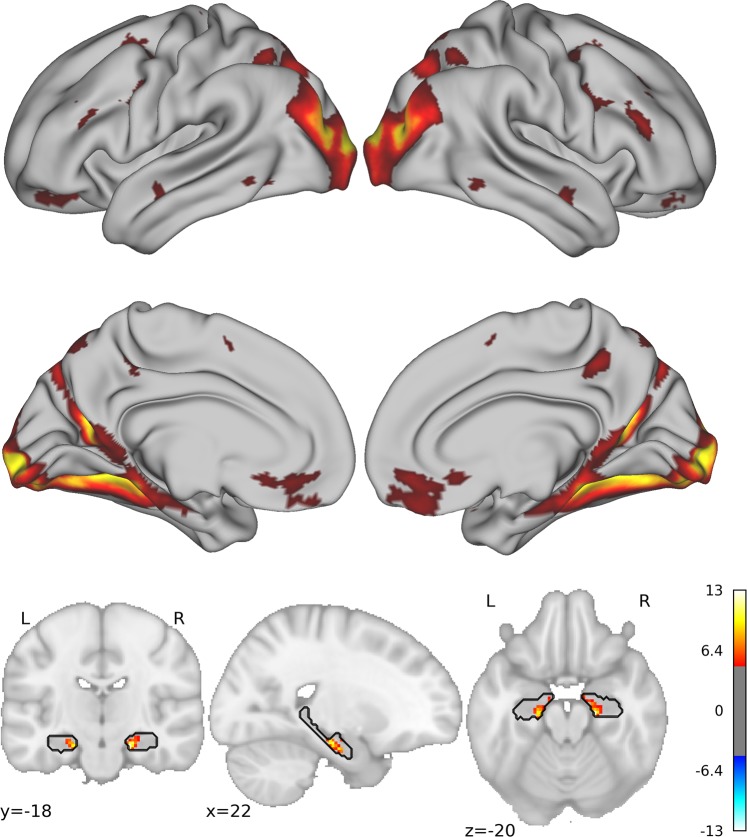
One-sample t-test for scenes>other (bodies, faces, tools) across whole brain, corrected for kinship. All active voxels are significant after family wise error correction (*P*_FWE_ < 0.05). T-statistic range = 4.8–56.8.

A one-sample *t*-test (accounting for kinship structure) across the final sample (*N* = 608) confirmed bilateral scene-related activity in the HC (Fig. 1). Critically, and consistent with previous work [15], significant voxels were located in the anterior-medial region within the hippocampus (*P*_FWE-WHOLE-BRAIN_ < 0.05) in response to scenes. Other regions including the retrosplenial cortex (RSC) and parahippocampal cortex (PHG) were also responsive to scenes in contrast to all other stimuli; faces, bodies, tools) as previous observed [15, 19, 20, 42, 48].

### Genetic effects of HC-BOLD during scenes > other

After quality control procedures, AD-PRS was regressed against average bilateral hippocampal BOLD (scene > other). After controlling for fixed effects (covariates) and familial confounds (random effects of familial environmental and genetic correlations), we identified a positive association between AD-PRS and HC scene BOLD (*β* = 0.102; *P* = 0.016). We observed similar associations at other liberal *p*-thresholds, but not conservative ones (Table 2). We observed no significant associations between performance (*n*-back accuracy) and *APOE* ɛ4 status or AD-PRS (*P* > 0 .05, in all cases).

**Table 2:** Linear regression models for hippocampal BOLD (arbitrary units) and volume (mm^3^).

		HC BOLD (a.u.)				HC volume (mm^3^)			
Model	SNPs	*β*	95% CI		*P*	*β*	95% CI		*P*
*APOE* + ɛ4	2	−0.064	−0.251	0.123	0.501	0.022	−0.132	0.176	0.776
pT_5 × 10^−8^	21	−0.032	−0.113	0.048	0.431	−0.041	−0.110	0.027	0.237
pT_0.001	737	0.013	−0.070	0.096	0.757	−0.054	−0.124	0.016	0.133
pT_0.005	2642	0.071	−0.011	0.153	0.088	−0.086	−0.156	−0.016	0.016
pT_0.01	4747	0.057	−0.026	0.140	0.178	−0.082	−0.152	−0.012	0.022
pT_0.05	17,297	0.073	−0.012	0.159	0.091	−0.032	−0.103	0.039	0.377
pT_0.1	29,718	0.073	−0.010	0.157	0.086	−0.004	−0.074	0.065	0.902
pT_0.2	49,658	0.092	0.009	0.175	0.029	−0.001	−0.071	0.070	0.987
pT_0.5	91,417	0.102	0.019	0.186	0.016	−0.012	−0.082	0.057	0.727
pT_1	133,305	0.104	0.020	0.187	0.015	−0.012	−0.082	0.057	0.725

Standardised beta reflects adjusted beta coefficients (controlling for genetic and demographic confounds) and 95% confidence intervals for each estimate. AD-PRS = Alzheimer’s disease polygenic risk score. ɛ4+ indicates the presence of an *APOE* ɛ4 allele. HC BOLD (a.u.) regressions also controlling for HC volume

### Negative control results

**Fig. 2:**
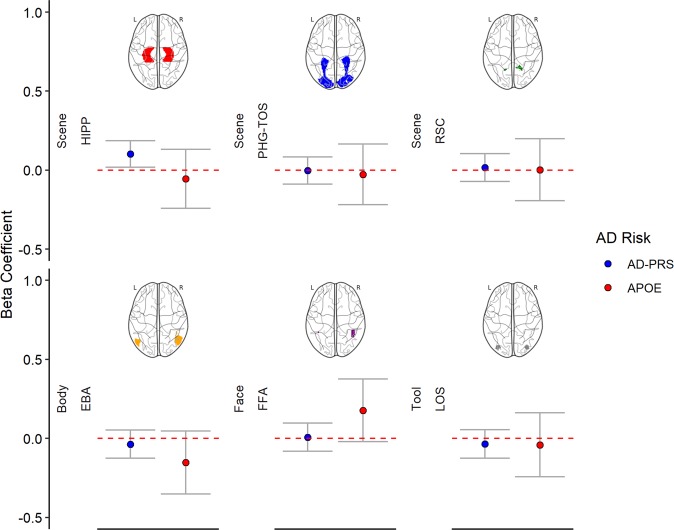
Negative control results: standardised beta-coefficients (*Y*-axis) for AD-PRS and *APOE* ɛ4 status (controlling for demographic and genetic confounds) for place selective cortical regions (top row: within hippocampus (HIPP); parahippocampal gyrus—transverse occipital sulcus (PHG-TOS); and the retrosplenial cortex (RSC). BOLD selective for bodies, faces and tools were assessed within the extra-striate body area (EBA); fusiform face area (FFA) and the lateral occipital cortex (LOC), respectively. Error bars reflect 95% confidence intervals of the beta coefficient. Figure includes +/−95% confidence intervals.

We repeated the linear-mixed effect model (as per Table 2) in (i) two other cortical regions associated with increased BOLD for scenes > other and (ii) three cortical regions associated with increased BOLD for bodies, faces and objects (EBA, FFA, LOC, receptively). We found no effect of AD-PRS or *APOE* ɛ4 status on any of these cortical regions (Fig. 2).

### Genetic effects in voxel-wise HC analysis

**Fig. 3:**
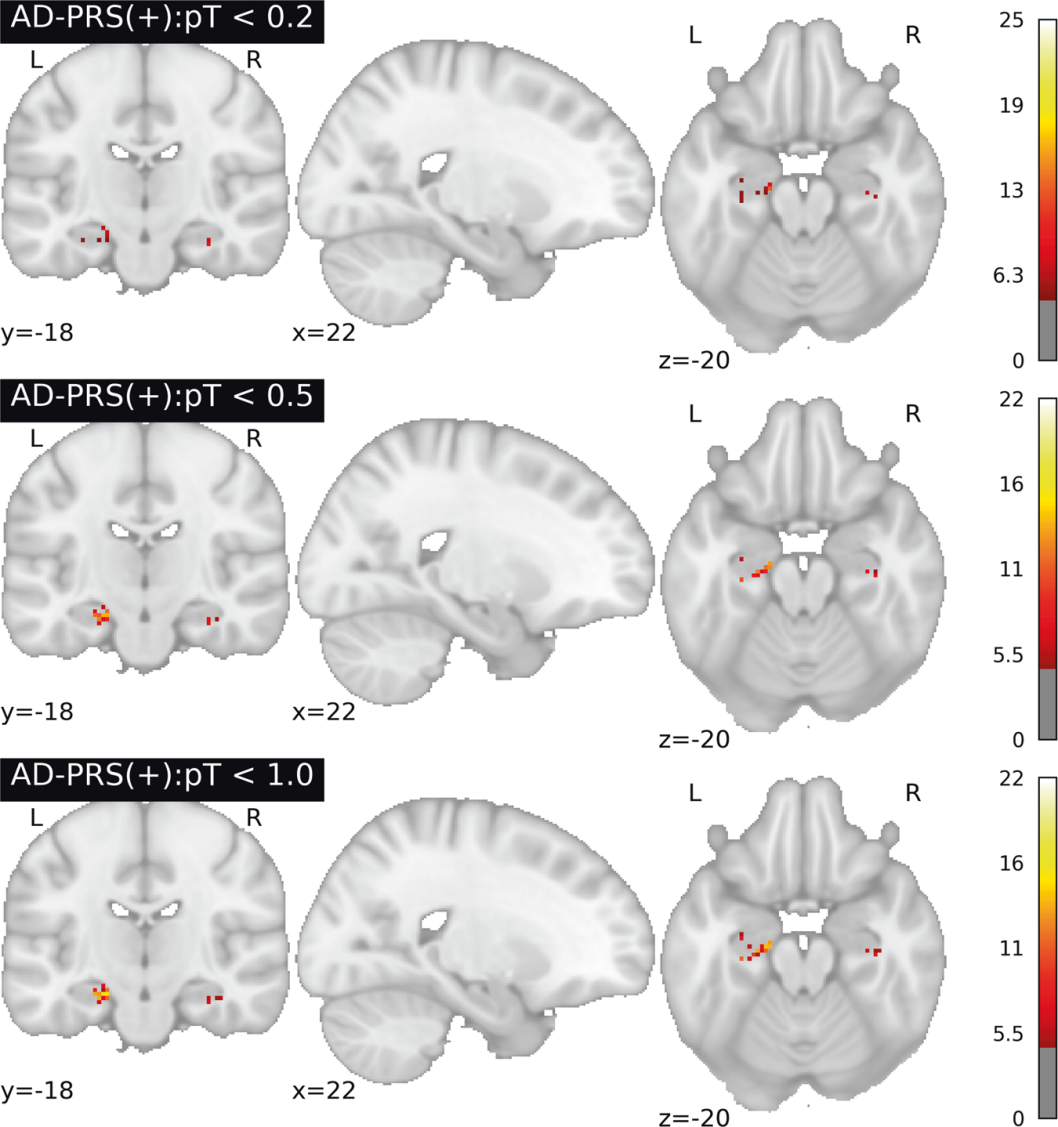
Positive association between AD-PRS and HC BOLD after controlling for demographic and genetic confounds. Voxel-wise search within the hippocampus show clusters within the right/left hippocampus that was significant after controlling for the family wise error (*P*_FWE-ROI_) using a threshold free cluster enhancement and accelerated permutation approach. Colour-bar represents *t*-statistic.

The voxel-wise search within the bilateral hippocampus identified bilateral clusters of voxels across the hippocampus that were significantly associated with AD-PRS after correction for the family-wise error. These voxels were proximal to grey matter within the anterior-medial HC (Fig. 3). We did not find any further brain-wide associations between AD-PRS or *APOE* and BOLD that survived family wise error correction.

## Discussion

We sought to explore whether common AD risk alleles (identified via GWAS) contribute to variation in HC BOLD during scene encoding in young healthy individuals. While several studies have linked AD-PRS to memory [49–51] and structural brain changes across the lifespan [52–54], this work is amongst the first to quantify the relationships between AD-PRS and functional brain activity, specifically via task-based fMRI in AD vulnerable regions.

More specifically, we used an embedded category localiser to target HC function in a large sample (*N* = 608), powered to detect relatively small effects. We identify a positive relationship between scene selective HC BOLD (a key node in the scene-processing network) and AD-PRS in young healthy individuals, decades before the onset of potential symptoms. We further demonstrate that this observation was (a) specific to the HC and (b) selective to scene processing. That is, no significant associations were observed between AD-PRS and brain activity for other functional contrasts (bodies, faces, tools) within their respective networks. This is one of the first studies to demonstrate an association between HC BOLD and AD-PRS in young individuals at such a scale.

Our findings support a hypothesis that the broader AD polygenic architecture may influence AD sensitive markers of brain function such as HC BOLD. Our findings are also in line with established theoretical models of AD where hyper-activation is observed in young cohorts at risk of developing AD leading to a steep and steady decline in brain function/connectivity as individual’s transition through Braak stages [23, 55]. While further investigation is required, we suggest that the positive association between AD-PRS and HC BOLD may reflect downstream mechanisms of neuronal hyper-activation. That is, the higher someone’s cumulative genetic risk for developing AD, the higher the neuronal hyper-activation. Our results also broadly conform to prior models that suggest the HC is susceptible and vulnerable to the pathogenesis in AD (12–14), providing support that HC function in individuals with increased AD risk is altered in early life processes. While we found no overt effects of AD genetic risk on task performance, we cannot exclude the possibility that altered hippocampal activity may reflect disruptions in other hippocampal-dependent cognitive processes such as navigation efficiency [56]. Future prospective studies that comprehensively assess modes of co-variation between hippocampal—dependent behaviour and brain function will help establish overt consequences of aberrant hippocampal BOLD. Our findings highlight that considerations about the broader polygenic architecture of AD should be explored to brain imaging studies of AD genetic risk.

While BOLD is considered an indirect measure of neuronal activity, it is a signal derived from a number of physiological processes and any disruption in vascular physiology and tissue oxygenation may pose a risk to neural responses during functional tasks. While we are unable to test this hypothesis within the current dataset, we suggest future studies focus on cerebrovascular response beyond BOLD to elucidate the precise physiological alterations that underpin task-related changes in AD risk. We suggest this hypothesis needs to be further assessed with more quantitative MRI methods to measure brain activity physiology more directly than BOLD. Our results may reflect an altered cerebral regulatory response to the scene-processing task in the HC or an alteration in metabolic function and oxygen supply/demand to the HC in response to scenes. Early evidence already suggests that there are alterations in task-dependent hippocampal cerebrovascular reactivity in young carriers of *APOE* ɛ4 [57]. Our preliminary data also suggests that AD-PRS may influence resting cerebral blood flow [30], warranting future studies linking AD-PRS to MRI parameters such as the cerebral metabolic rate of oxygen (CMRO2) and oxygen extraction fraction (OEF) to establish the regional, molecular impact of AD-PRS on cerebro-vasculature and/or brain metabolism.

We did not identify any association between *APOE* ɛ4 status and scene encoding in the HC or any other perceptual stimulus category (bodies, faces, tools) in their respective network. We therefore suggest that scene selective HC BOLD is not associated with presence/absence of the *APOE* ɛ4 allele in young healthy individuals but rather associated with the cumulative impact of common AD risk genes identified via GWAS. Our voxel wise analysis of AD-PRS within the HC revealed a bilateral cluster within the region of the anterior-medial HC for in response to the scenes more than any other category contrast of stimulus (tools, bodies, faces). High-resolution 7 T MRI has identified anterior-medial subunits that are thought to be more involved in scene encoding than others, namely the subiculum [58]. The subiculum has emerged as a key region of the HC hypothesised to be most susceptible to pathology when transitioning from mild cognitive impairment (MCI) to AD [59]. The subiculum regions may therefore be most susceptible to alterations when assessing the polygenic impact on task specific scene encoding in the HC and may be a useful functional marker to assess HC changes in those with increased risk for developing AD. However, it is important to note that 3 T MRI is not optimal to dissociate between HC subfields at this level, therefore we suggest that future work aims to reproduce these preliminary findings with a more HC focused high-resolution fMRI sequence at 7 T.

Our findings should also be considered in light of the following limitations. First, we note that the YA-HCP sample is ethnically heterogeneous. While we minimise population effects by (i) excluding non-Caucasian participants and (ii) including 20 genetic principle components as covariates, we cannot fully reject population stratification effects. Second—while the pedigree structure was modelled (via kinship matrix and exchangeability blocks), we cannot fully exclude residual kinship structure that may influence our results. We also note that we only observed associations between AD-PRS and HC BOLD at the liberal *P*-thresholds that capture maximal variability in AD risk models [29]. This would suggest that our association was explained by the cumulative effects of thousands of low confidence alleles (estimated at liberal p-thresholds), rather than well-established AD risk alleles (such as those that surpass GWAS significance). While this is consistent with the polygenic model of complex traits [60], it may implicate a role for complex polygenic traits that share a genetic architecture with AD such as neural (e.g., cognitive ability, educational attainment [2]) and/or vascular process (e.g., blood plasma lipid and cholesterol metabolism [61]). Future genetic tools will help to refine future polygenic approaches to separate genetically correlated heritable traits [62].

In conclusion, our findings suggest that the cumulative impact of a large combination of AD risk alleles is associated with altered HC BOLD during scene encoding, which may predispose future risk to AD. The observations provide additional evidence that genetic risk for AD manifests decades before the onset of symptoms, in medial temporal lobe structures expressing the earliest molecular evidence of pathology. Our findings suggest task-dependent hippocampal BOLD may be useful in elucidating potential therapeutic and interventional strategies that may aid in detection and prevention of AD.
